# A Hospital Lead

**Published:** 1920-03-13

**Authors:** 


### A Hospital Lead.

A valuable step in the interests of children has been taken by the Committee of Management of the Great Northern Central Hospital. In order to meet the growing needs of North London, they have provided, as from the first of this month, a special department for diseases of children, and a child welfare consultative centre. There are two sections—medical and surgical—and the session is held on Monday afternoons. The department is intended for children under twelve years of age, and medical and surgical specialists are in charge. References on forms specially provided can be made from any of the child welfare centres in London, and the specialists' opinion on cases so referred is sent to the centre concerned.

Every effort is being made to encourage closer personal working arrangements between the hospital and the various centres, and it is hoped that this new service will meet a want that is very great. In addition to this, the same hospital has recently established an ante-natal clinic, and cases are similarly referred to the gynæcological department of the hospital from district maternity centres. If people realised a little more fully what a large part the hospitals are playing in this as in so many other directions, there would be much less necessity for the continued discussion of the problem of means of subsistence for these great institutions.

